# Frailty has a stronger association with inflammation than age in older veterans

**DOI:** 10.1186/s12979-016-0082-z

**Published:** 2016-10-19

**Authors:** P. Van Epps, D. Oswald, P. A. Higgins, T. R. Hornick, H. Aung, R. E. Banks, B. M. Wilson, C. Burant, S. Graventstein, D. H. Canaday

**Affiliations:** 1 Geriatric Research, Education, and Clinical Center, Louis Stokes Cleveland VA Medical Center, Cleveland, OH USA; 2 Division of Infectious Disease, Case Western Reserve University, Cleveland, OH USA; 3 Division of Geriatrics, Department of Medicine, Case Western Reserve University, Cleveland, OH USA; 4 School of Nursing, Case Western Reserve University, Cleveland, OH USA

**Keywords:** Inflammatory index, Cytokines, Functional decline and coagulation

## Abstract

**Background:**

Upregulation of pro-inflammatory cytokines has not only been associated with increased morbidity and mortality in older adults but also has been linked to frailty. In the current study we aimed to compare the relative relationship of age and frailty on inflammation and thrombosis in older veterans.

**Results:**

We analyzed 117 subjects (age range 62–95 years; median 81) divided into 3 cohorts: non-frail, pre-frail and frail based on the Fried phenotype of frailty. Serum inflammatory markers were determined using commercially available ELISA kits. Frail and pre-frail (PF) subjects had higher levels than non-frail (NF) subjects of IL-6 (NF vs. PF: *p* = 0.002; NF vs. F: *p* < 0.001), TNFR1 (NF vs. F: *p* = 0.012), TNFRII (NF vs. F: 0.002; NF vs. PF: *p* = 0.005) and inflammatory index: = 0.333*log(IL-6) + 0.666*log(sTNFR1) (NF vs. F: *p* = 0.009; NF vs. PF: *p* < 0.001). Frailty status explained a greater percent of variability in markers of inflammation than age: IL-6 (12 % vs. 0.3 %), TNFR1 (5 % vs. 4 %), TNFR2 (11 % vs. 6 %), inflammatory index (16 % vs. 8 %). Aging was significantly associated with higher fibrinogen (*p* = 0.04) and D-dimer levels (*p* = 0.01) but only among NF subjects.

**Conclusion:**

In conclusion, these data suggest that among older veterans, frailty status has a stronger association with inflammation and the inflammatory index than age does. Larger studies, in more diverse populations are needed to confirm these findings.

## Background

The paradoxical phenomena of decline in immune function or immunosenescence and increased inflammation are well described in aging. Immune dysregulation in older adults results in imbalance between pro and anti-inflammatory cytokines and consequently a low-grade chronic inflammatory state [1, 2]. Upregulation of cytokines such as interleukin-1 (IL-1), interleukin-6 (IL-6) and tumor necrosis factor-α (TNF-α) that contribute to systemic inflammation have been independently associated with increased morbidity and mortality in older adults [3, 4]. It is now also well accepted that aging is associated with markers of activated coagulation, contributing to an overall pro-inflammatory state [5–7]. The term “inflammaging” refers to this inflammatory state and its association with age-associated diseases [8].

Besides the increased risk of morbidity, chronic inflammation has also been linked to functional decline in older adults and has been theorized as a potential explanation for the biologic basis of frailty [9, 10]. In this study, frailty is defined according to Linda Fried’s phenotype as a biologic syndrome of decreased reserve and decreased resistance to stressors that is a strong marker for poor health outcomes including falls, disability and death [11]. There is growing link between frailty and inflammation. Elevated serum levels of IL6, TNF-α, and C-reactive protein (CRP) have been linked with poor function and mobility status [9, 12]. Elevated levels of IL-6 have been associated with slower gait velocity and are predictive of gait speed decline in community-dwelling older adults [13]. Higher plasma IL-6 levels have also been seen in older adults with performance deficits in activities of daily living (ADLs) than those without any functional deficits [14]. Similarly, markers of coagulation and endothelial dysfunction have been linked to frailty and associated with poorer outcomes. D-dimer and other markers of activated coagulation have been associated with limitation in a wide variety of functional domains, including independent activities of daily living [15, 16]. Frail and pre-frail subjects from the Cardiovascular Health Study had significantly higher levels of fibrinogen, factor VIII, and D-dimer compared with the non-frail group [17]. Frail adults are also at increased risk of venous thromboembolism when compared with non-frail persons of the same age [18]. Despite the mounting evidence for role of inflammation and coagulation in aging and frailty, measurements of inflammatory markers have not yet been incorporated into standard clinical practice, partly because a “gold standard” to reliably predict incident adverse outcomes in older adults is needed. More recently, inflammatory index, an additive index of serum IL-6 and soluble TNF-α receptor −1 (TNFR1) has been shown not only to best capture age-associated chronic inflammation but also predict mortality in older adults [19]. There are limited data regarding the inflammatory index in the context of frailty. For that matter, data on the relative association of age and frailty on inflammation and coagulation in older adults is also lacking. In the present study, we examined how age and frailty are related to an expanded set of inflammatory and coagulation markers.

## Methods

### Study subjects

We enrolled 117 veteran subjects, 60 years of age or older (age range 62–95 years; median 81), receiving care at the Louis Stokes Cleveland Veteran Affairs Medical Center (LSCVAMC) outpatient clinics for this study. We also enrolled 25 subjects, which included veteran and non-veteran subjects, younger than 60 (age range 22–54, median 39). Subjects receiving immunosuppressive medications or with immunosuppressive conditions including HIV, cancer undergoing chemotherapy, severe anemia were excluded from the study. Each subject underwent a blood draw for the measurements of inflammatory and coagulation markers. Informed consent was obtained from all participants. Human experimentation guidelines of the Department of Health and Human Services were followed in the conduct of this study. The study was reviewed and approved by Institutional Review Boards at the LSCVAMC and Case Western Reserve University.

### Frailty measurement

The Fried’s frailty assessment tool, a widely accepted and validated instrument of frailty measurement in older adults, was utilized for functional assessments [11]. Study staff administered the five components of Fried frailty assessment tool: weakness as assessed by grip strength (measured by Jamar dynamometer average of 3 trials with dominant hand), walking speed (15 feet, straight line, one way), unintended weight loss > = 10 pounds in the past year, self-reported exhaustion (two Likert-type questions from CES-D Depression Scale [20]), and physical activity based on the Minnesota Leisure Time activity questionnaire [21]. Grip strength was stratified by sex and body mass index, walking speed was stratified by sex and height and physical activity score was stratified by sex as suggested by Fried and colleagues. Subjects were assigned to the non-frail (NF) category if for 0 criteria met, pre-frail (PF) for 1–2 criteria and frail (F) for 3 or more criteria met on the Fried frailty instrument.

### Biomarker measurements

Serum and soluble inflammatory and coagulation markers were obtained using commercially available kits. Assays were performed according to the manufacture’s protocols. The following markers were measured in blood: pro-inflammatory cytokines serum IL-6 (Human IL-6 Qunatikine HS Elisa Kit), serum IL-18 (Human IL-18/IL-1 F4 ELISA), soluble TNF-α receptor-1 (TNFR1) (Human sTNF RI/TNFRSF1A Quantikine ELISA Kit), and soluble TNF-α receptor-2 (TNFR2) (Human sTNF RII/TNFRSF1B Quantikine ELISA Kit), Interferon gamma-induced protein-10 (IP-10)(Human CXCL10/IP-10 Quantikine ELISA Kit), soluble CD14 (Human CD14 Quantikine ELISA Kit) (all R&D System, Minneapolis, MN, USA) and pro-inflammatory protein C-reactive protein (CRP) (EIA kit, Cayman Chemical Company, Ann Arbor, MI, USA), Serum Amyloid A (SAA) (ELISA, Assaypro, St. Charles, MO, USA) and were also measured. Inflammatory index is a calculated parameter: 0.333*log (IL-6) + 0.666*log (sTNFR1). The following markers of thrombosis and coagulation were also measured: Plasminogen activator inhibitor-1 (PAI-1) (AssayMax Human PAI-1 ELISA kit, Assaypro, St. Charles, MO, USA), D-dimer (ELISA ZYMUTEST DDimer, ANIARA, West Chester, OH, USA) and Fibrinogen (immunoperoxidase assay, GenWay, San Diego, CA, USA).

### Statistical analysis

Analysis was completed in SPSS; Graphpad Prism 6.0 and R 3.2.2 using the *ggplot2* packages were used to graphically represent the data. ANOVA was used to compare means of various biomarkers between frailty groups. In cases where the frailty F statistic suggested a significant overall affect, post hoc pairwise comparisons were performed with p value adjustment with Bonferroni or Tamhane, as determined by homogeneity of variance. Associations between age and each biomarker measured were examined using Spearman rank correlations. Log transformed values were used to represent correlations with frailty groups. The Spearman Rank correlation is robust to transformations and outliers. These correlations were also examined within groups of frailty. The explained variability in biomarkers, as measured by R-squared values, obtained from Pearson correlations for age and ANOVA for frailty group, was compared between age and frailty. The R-squared statistic measures the percent of variability in an outcome explained by one or more covariates. For a single continuous covariate, it can be calculated as the square of the Pearson correlation coefficient; for a single categorical variable, it can be calculated as the regression sum of squares due divided by the total sum of squares as summarized in typical ANOVA results. Thus, comparing the squared Pearson correlation coefficient to the ANOVA-based R-squared allows the percent of variability in an outcome explained by a categorical variable and a continuous variable to be identically measured [22]. For patients with available outpatient data from the two years prior to enrollment, we also obtained outpatient diagnosis codes from the electronic database and calculated the Charlson comorbidity index using a validated list of diagnosis codes [23]. The index was calculated, without the addition of points for age, for 103 subjects (mean = 2.75, range = 0 to 8). We performed regression analyses comparing the explained variability using comorbidity and age vs. comorbidity and frailty.

## Results

The one hundred and seventeen subjects over age 60 were divided into 3 cohorts based on the Fried frailty measurement categories: non-frail (*N* = 23), pre-frail (*N* = 50) and frail (*N* = 44). The median age in NF group (68 years, range 62–90) was significantly lower than the PF (80 years; 62–92) and frail (82 years; 62–92) groups (*p* < 0.005 for both). As expected for an older veteran population, majority of the subjects were male (96 %). African Americans accounted for over half (54 %) of the study subjects.

### Markers of inflammation and the inflammatory index among frailty groups

**Fig. 1:**
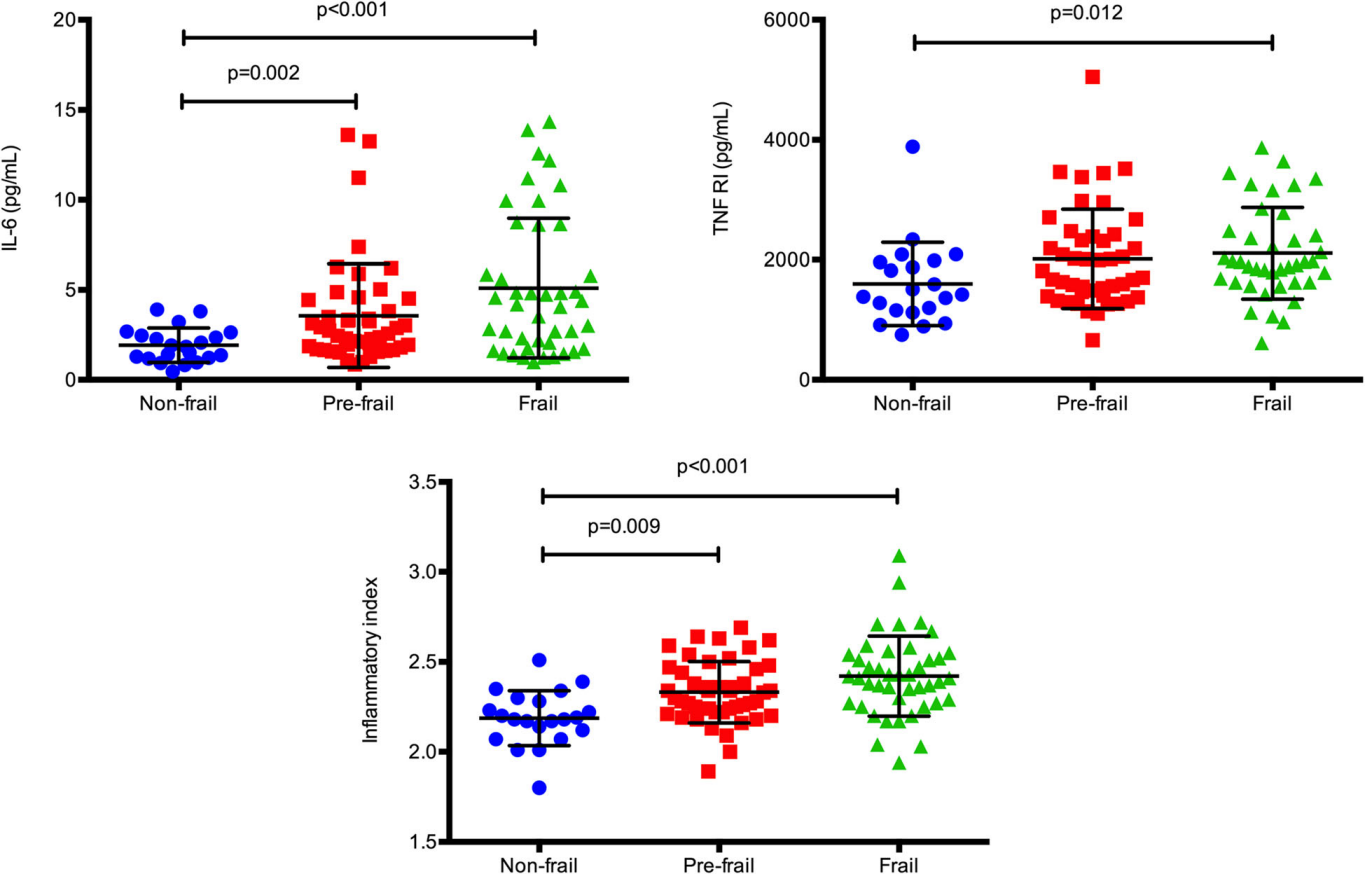
Inflammatory index and related markers among frailty groups. Comparisons are made using ANOVA between non-frail (circles), pre-frail (squares) and frail (triangles) subjects as determined by the Fried’s Frailty assessment tool. In cases where the frailty F statistic suggested a significant overall affect, post hoc pairwise comparisons were performed with p value adjustment with Bonferroni or Tamhane, as determined by homogeneity of variance. Only significant p values are marked. Inflammatory index is a calculated parameter: 0.333*log (IL-6) + 0.666*log (sTNFR1)

**Fig. 2:**
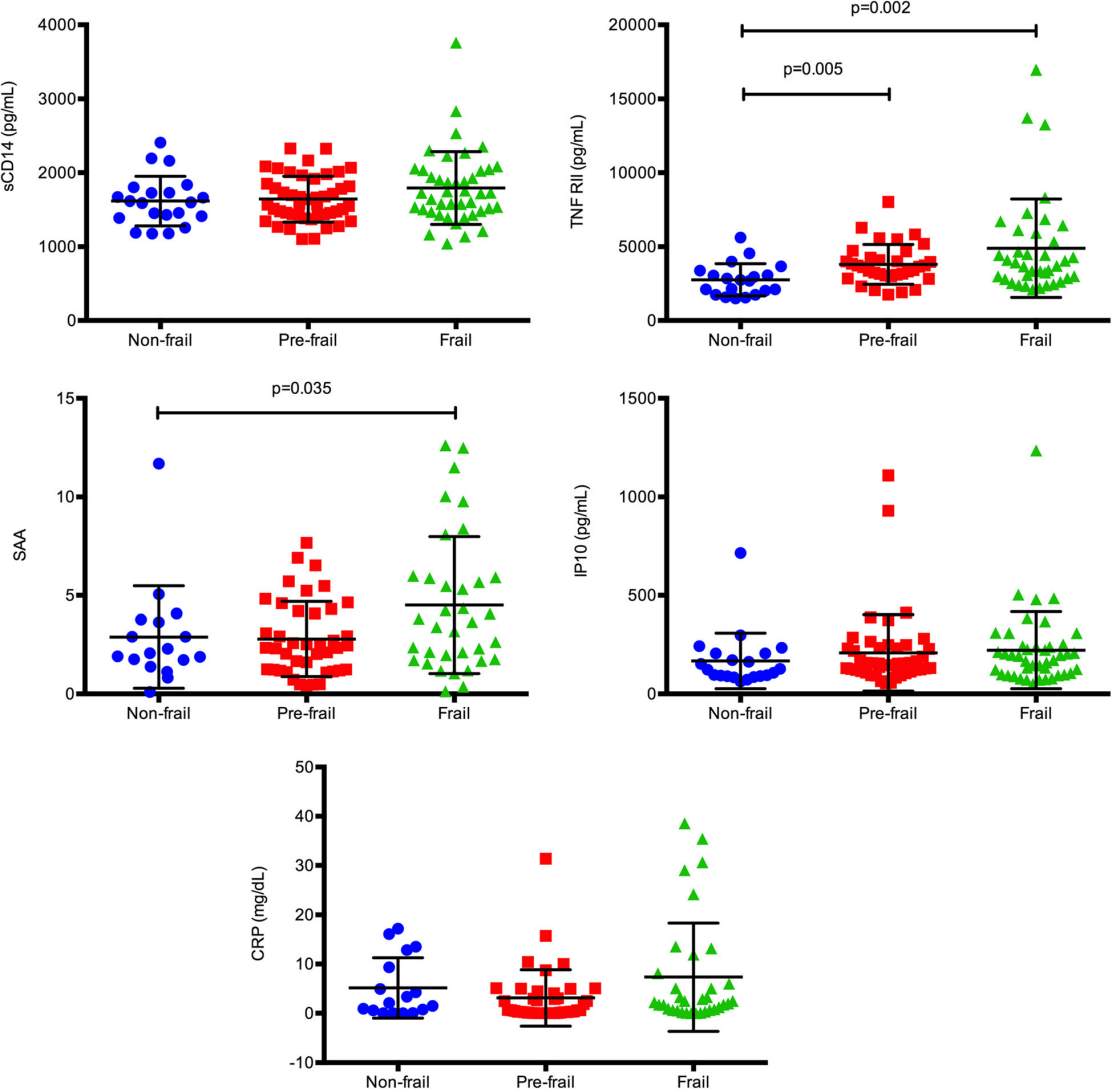
Markers of inflammation among frailty groups. Comparisons are made using ANOVA between non-frail (circles), pre-frail (squares) and frail (triangles) subjects as determined by the Fried’s Frailty assessment tool. In cases where the frailty F statistic suggested a significant overall affect, post hoc pairwise comparisons were performed with p value adjustment with Bonferroni or Tamhane, as determined by homogeneity of variance. Only statistically significant p values are marked

We compared all the markers of inflammation tested among frailty groups. Frail and pre-frail subjects had higher levels of markers incorporated into the Inflammatory Index as well as a higher calculated Inflammatory Index levels (Fig. 1). Mean IL-6 levels among frail subjects were nearly three times those found in NF subjects (1.88 pg/mL vs. 5.09 pg/mL; *p* < 0.001) and nearly twice as high among PF subjects compared with NF subjects (1.88 vs. 3.56; *p* = 0.002) than non-frail subjects. Similarly TNFR1 levels were significantly higher among frail subjects compared with NF subjects (1577 pg/mL vs. 2624 pg/mL; *p* = 0.012). Inflammatory index levels were higher in both PF and frail groups compared with NF group (NF vs. PF: *p* < 0.001; NF vs. F: *p* = 0.009). An additional marker that was significantly elevated in both the PF and frail groups was TNFRII (NF vs. F: 0.002; NF vs. PF: *p* = 0.005; Fig. 2). The mean level of SAA was significantly higher in frail group compared with NF group (2.8 vs. 4.5; *p* = 0.035). Notably there were no differences in markers of inflammation detected between PF and frail subjects. We also did not find any significant differences among frailty groups between sCD14, IP10, CRP and IL-18 levels (Fig. 2).

### Frailty explains the variability in markers of inflammation not age

**Fig. 3:**
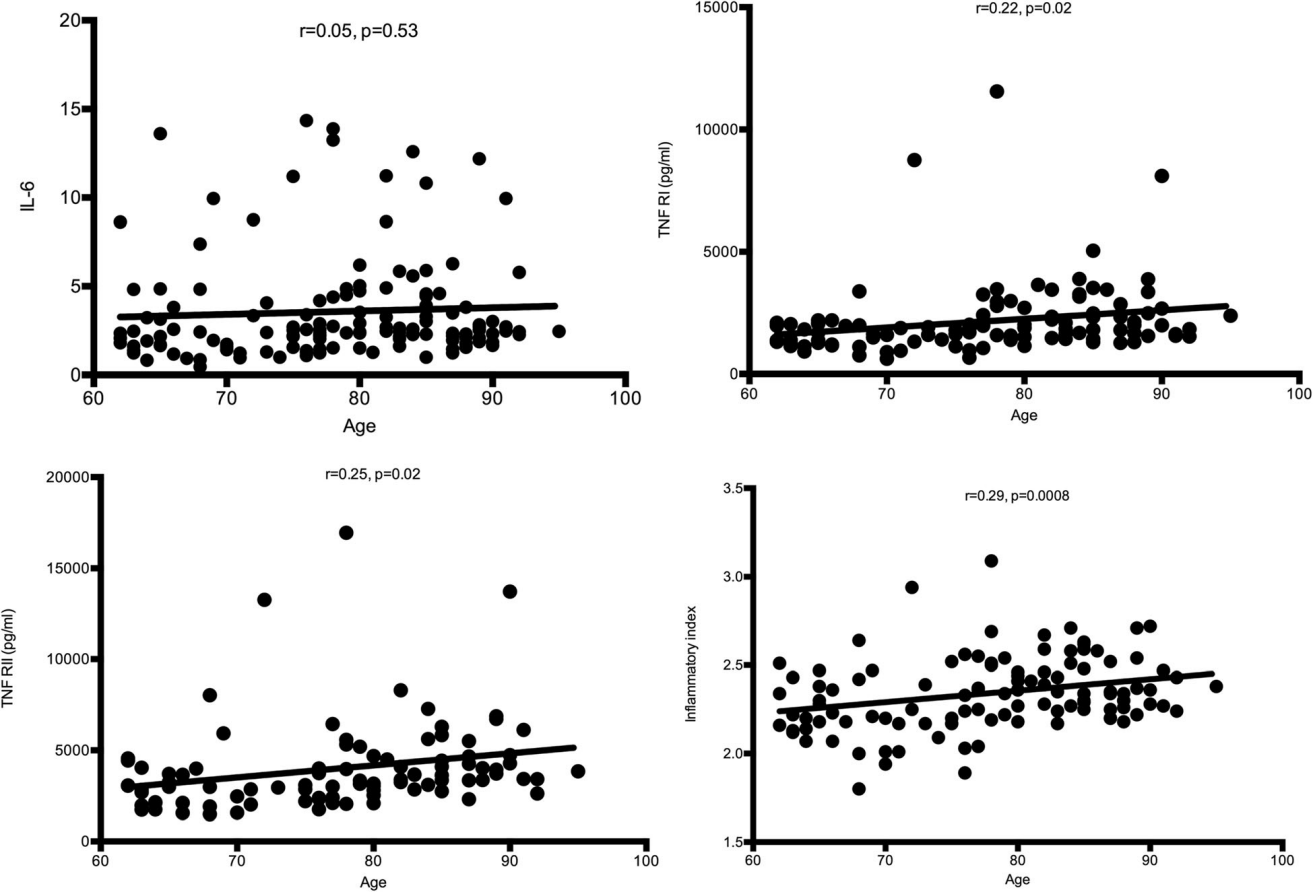
Spearman Rank correlations between age and markers of inflammation. The three frailty groups are combined for these comparisons

When all the subjects were grouped together, using Spearman Rank correlations, age was positively associated with TNFR1 (*r* = 0.22; *p* = 0.02), TNFR2 (*r* = 0.25; *p* = 0.02) and the inflammatory index (*r* = 0.28, *p* = 0.008) but not IL-6 (*r* = 0.05; *p* = 0.5) (Fig. 3). No other markers tested correlated with age in the cohort of patients over the age of 60 years (data not shown). When correlations were tested within frailty status groups however, there were no statistically significant associations between age and markers of inflammation (data not shown). In order to determine whether frailty status or age had a greater association with markers of inflammation, the R^2^ values from ANOVA were compared with R^2^ from spearman rank correlations. Frailty status explained a greater percent of variability in markers of inflammation than age: IL-6 (12 % vs. 0.3 %), TNFR1 (5 % vs. 4 %), TNFR2 (11 % vs. 6 %), inflammatory index (16 % vs. 8 %). We did not find any significant interactions between age and frailty among the markers of inflammation and coagulation tested. For the subset of patients for which we had data available to calculate Charlson comorbidity index, we performed regression analyses controlling for comorbidity. After controlling for comorbidity, frailty explained more of the variability compared with age in IL-6 levels (12 % vs. 1.5 %), inflammatory index (18 % vs. 15 %), CRP (8 % vs. 3 %), SAA (12 % vs. 1 %) sCD14 (8 % vs. 7 %). On the other hand, after controlling for comorbidity, age was slightly better at explaining variability in TNFR1 (18 % vs. 12 %) and TNFR2 (18 % vs. 16 %). We also examined relationship between CMV seropositivity and various inflammatory markers, however the vast majority of our subjects were CMV+ (76 %) and we did not detect such a relationship (data not shown).

### Coagulation markers, frailty and age

**Fig. 4:**
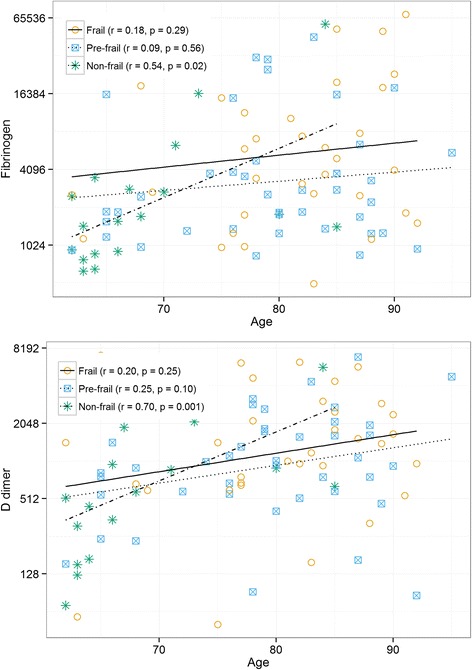
Spearman Rank correlations between age and markers of coagulation within frailty groups. Corresponding r and p values are listed for each frailty group non-frail (star with dashed line), pre-frail (square with dotted line), frail (open circle with continuous line)

Fibrinogen, PAI-1 and D-dimer levels were not significantly different among frailty groups. Age was positively associated with both fibrinogen (*r* = 0.21; *p* = 0.04) and D-dimer (*r* = 0.27; *p* = 0.01) (data not shown). However, within frailty status groups, the only statistically significant associations between age and coagulation markers were seen in the NF group. Both D-dimer (*r* = 0.7, *p* = 0.001) and Fibrinogen (*r* = 0.54; *p* = 0.02) were strongly positively associated with age in this group (Fig. 4). Overall, age explained a greater percent of variability in fibrinogen (4.4 % vs. 3.9 %) and D-dimer (7 % vs. 4 %) than frailty status. After controlling for comorbidities using Charlson score in a regression analysis, age explained more variability in both D-dimer (11 % vs. 2 %) and fibrinogen (7 % vs. 6 %).

## Discussion

In the present study we sought to determine the relative effect of age compared with frailty on markers of inflammation and coagulation in older adults. We observed significant elevations in pro-inflammatory cytokines as well as the inflammatory index among older adults who were pre-frail or frail. While age was associated with some of the markers studied, in particular the inflammatory index, these associations were not present within frailty groups. Additionally we also observed that the variability in inflammatory markers was explained to a much greater extent by frailty status than age. Coagulation markers fibrinogen and D-dimer, on the other hand, were not significantly different among frailty groups but did correlate with age. These correlations persisted among those with intact functional status. Age appeared to explain the variability in coagulation markers more than frailty.

Similar to prior studies we found elevations in IL-6, SAA, TNFR1 and TNFR2 levels among those with functional decline [4, 12, 14, 24, 25]. A measure validated in older adults, the inflammatory index, which incorporates two of these markers, has recently been shown to be independently associated with frailty among aging HIV-infected and uninfected injection drug users [26]. We also found the inflammatory index scores to be significantly increased in frail and pre-frail older adults. Interestingly, unlike a previous report where the greatest differences between IL-6, TNF-α and CRP levels were seen between the pre-frail and frail adults [27], we did not see any significant differences between PF and frail subjects. In our cohort, pre-frail inflammatory profile more closely resembled that of frail subjects than those that were still functionally intact. While both the PF and frail groups were significantly older than NF group, we showed that in fact age did not explain variations in inflammatory markers as much as frailty status. This finding may indicate that even before functional decline becomes clinically apparent, the pro-inflammatory phenotype has already been set in motion.

Age was associated with increased TNFR1 and TNFR2, but unlike previously cited studies we did not find IL-6 or CRP levels to be significantly associated with age in this cohort over 60 years of age. The data presented in this study focused on those over the age of 60. When subjects of all ages were included in the analysis, IL-6 did correlate with age. Aging was associated with increasing inflammatory index score in our study, as has previously been shown [19]. However, age was not associated with the inflammatory index, or any other markers, when frailty groups were examined separately. This finding was confirmed by comparing the percentage of variability in markers explained by age vs. frailty. Frailty status had consistently greater association with inflammation than did age. Interestingly, this trend seemed to persist for most inflammatory markers, particularly IL-6, inflammatory index and CRP, even after controlling for comorbid conditions. This suggests that while chronic inflammation is a feature of aging looking over the entire age span, the inflammatory milieu is closely linked with frailty such that irrespective of chronologic age, frailty phenotype maybe a stronger predictor of chronic inflammation. However, as with other cross-sectional studies demonstrating association between frailty and inflammation, our study provides no insights into causality. It has been hypothesized that chronic inflammation may be the driving force behind functional decline and may form the biologic basis of age-associated conditions including frailty [28, 29]. Elevated levels of IL-6 have been linked to multiple age-associated conditions, such as atherosclerosis [30], dementia [31] and frailty [32], however causality and pathogenesis is yet to be proven. It is also plausible that increased levels of inflammatory cytokines maybe a compensatory mechanism in frailty and other age-associated conditions. Inflammatory response maybe triggered by chronic viral infections such as cytomegalovirus or other herpes viruses [33]. We were unable to link CMV chronic infection to inflammation due to very low numbers of seronegative subjects in our study. It is also possible that markers of inflammation are simply a byproduct of another causal mechanism of frailty such as excessive oxidative stress. Frailty in older adults has been associated with superoxide anion overproduction by nicotinamide adenine dinucleotide phosphate-oxidase (NADPH) oxidase and low-grade chronic inflammation [2]. Biomarkers of oxidative stress have also been associated with frailty in the Framingham Offspring Study, suggesting oxidative stress as the underlying mechanism contributing to frailty [34]. Whether targeted interventions at preventing functional decline may result in an improved pro and anti-inflammatory balance, regardless of age of the patient remains to be established.

We also tested the associations between frailty and age among markers of coagulation. In the present cohort there were no differences between D-dimer and fibrinogen levels among frailty groups. Prior studies have linked D-dimer and other markers of activated coagulation with limitation in functional abilities [4, 35]. It has been proposed that aging represents a pro-thrombotic state and that some of the markers of coagulation and thrombosis begin to rise early during the process of aging, suggesting a potential usefulness as markers for frailty [3]. We did find aging to be not only associated with both these coagulation markers but also more strongly associated with a pro-thrombotic state than frailty status. This association was present even after controlling for comorbid conditions. Surprisingly, when age was examined within frailty groups, the association remained very strong but only among non-frail subjects. This suggests that while aging results in a pro-thrombotic state, its effect maybe less pronounced in those who already have evidence of functional decline.

This study not only provides further evidence of association between frailty and inflammation but also demonstrates that chronological age maybe less important than functional ability when it comes to chronic inflammation among older adults. Further studies are not only needed to establish the nature of this association but also to investigate the effects of interventions to prevent functional decline or modulate the inflammatory cytokines in an effort to prevent age-associated conditions.

## Conclusion

In conclusion, among older adults, these data suggest that frailty may be more strongly related to inflammation and the inflammatory index than age. Coagulation factors, on the other hand appear to have a stronger association with chronological age than frailty. These findings warrant further investigation in larger, more diverse populations.

## Abbreviations

ANOVA: Analysis of variance
CMV: Cytomegalovirus
CRP: C reactive protein
EIA: Enzyme immunoassay
ELISA: Enzyme-linked immunosorbent assay
IL-1: Interleukin-1
IL-6: Interleukin-6
IP-10: Interferon gamma-induced protein-10
LSCVAMC: Louis Stokes Cleveland Veteran Affairs Medical Center
NADPH: Nicotinamide adenine dinucleotide phosphate-oxidase
NF: Non-frail
PAI-1: Plasminogen activator inhibitor-1
PF: Pre-frail
SAA: Serum Amyloid A
TNFR1: Tumor necrosis factor-α receptor 1
TNFR2: Tumor necrosis factor-α receptor 2
TNF-α: Tumor necrosis factor-α.
