# Erratum to: Frailty has a stronger association with inflammation than age in older veterans

**DOI:** 10.1186/s12979-017-0086-3

**Published:** 2017-02-16

**Authors:** P. Van Epps, D. Oswald, P. A. Higgins, T. R. Hornick, H. Aung, R. E. Banks, B. M. Wilson, C. Burant, S. Gravenstein, D. H. Canaday

**Affiliations:** 1 Geriatric Research, Education, and Clinical Center, Louis Stokes Cleveland VA Medical Center, Cleveland, OH USA; 2 Division of Infectious Disease, Case Western Reserve University, Cleveland, OH USA; 3 Division of Geriatrics, Department of Medicine, Case Western Reserve University, Cleveland, OH USA; 4 School of Nursing, Case Western Reserve University, Cleveland, OH USA

## Erratum

In the original publication of this article [1], the author S. Graventstein was listed incorrectly. The correct spelling should have been S. Gravenstein.
